# Biomechanical analysis of wrapping of the moderately dilated ascending aorta

**DOI:** 10.1186/s13019-015-0299-5

**Published:** 2015-08-01

**Authors:** Tomasz Plonek, Bartosz Rylski, Andrzej Dumanski, Przemyslaw Siedlaczek, Wojciech Kustrzycki

**Affiliations:** 1 Department of Cardiac Surgery, Wroclaw Medical University, Borowska 213, 50-556 Wroclaw, Poland; 2 Heart Centre Freiburg University, Freiburg, Germany; 3 MESco, 42-600 Gornicza 20A, Tarnowskie Gory Poland

**Keywords:** Aneurysm, Aorta, Wrapping

## Abstract

**Background:**

External wrapping is a surgical method performed to prevent the dilatation of the aorta and to decrease the risk of its dissection and rupture. However, it is also believed to cause degeneration of the aortic wall. A biomechanical analysis was thus performed to assess the stress of the aortic wall subjected to external wrapping.

**Methods:**

A stress analysis using the finite elements method was carried out on three models: a non-dilated aorta, a moderately dilated aorta and a wrapped aorta. The models were subjected to a pulsatile flow (120/80 mmHg) and a systolic aortic annulus motion of 11 mm.

**Results:**

The finite elements analysis showed that the stress exerted on the outer surface of the ascending aorta in the wrapping model (0.05–0.8 MPa) was similar to that observed in the normal aorta (0.03–0.7 MPa) and was lower than in the model of a moderately dilated aorta (0.06–1.4 MPa). The stress on the inner surface of the ascending aorta ranged from 0.2 MPa to 0.4 MPa in the model of the normal aorta, from 0.3 to 1.3 MPa in the model of the dilated aorta and from 0.05 MPa to 0.4 MPa in the wrapping model.

**Conclusions:**

The results of this study suggest that the aortic wall is subjected to similar stress following a wrapping procedure to the one present in the normal aorta.

## Background

The dilatation of the tubular part of the ascending aorta is often diagnosed in patients with aortic valve pathology. Some surgeons prefer the ”watch and wait” approach to a moderately dilated aorta accompanying aortic valve disease. However, this approach carries the risk of a reoperation in case of further aortic dilatation. Thus, most cardiac surgeons choose to replace the dilated tubular part of the ascending aorta or perform other surgical procedures like aortoplasty or wrapping.

In the 1970’s, Robicsek et al. published the results of a theoretically less invasive surgical technique, called an aortoplasty [1]. It involved the removal or plication of the excessive aortic wall and the restoration of the normal aortic diameter. In some patients, an additional external wrapping was performed to reinforce the segment of the aorta subjected to aortoplasty and to prevent it from redilatation. Gill and Dunning analyzed the results of an aortoplasty with and without concomitant wrapping [2]. They found that aortoplasty had relatively good postoperative results with low early morbidity and mortality. Still, leaving a defective aortic wall without an additional reinforcement may carry the risk of redilatation. Adding external reinforcement, called aortic wrapping could strengthen the aortic wall. This external corset creates a barrier which should prevent the aorta from redilatation. Some studies have presented good mid-term and long-term results of wrapping without concomitant aortoplasty (isolated wrapping) [3–7]. In this technique, the dilated aorta is not only wrapped with the vascular prosthesis but also squeezed to restore its normal diameter. Although the results of isolated wrapping seem to be promising [8], some surgeons regard this technique as potentially dangerous as it may lead to aortic wall degeneration [9–11].

**Fig. 1:**
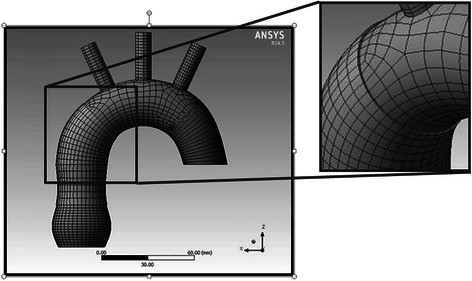
The discrete model of the aortic wrapping with visible finite elements’ net

There is some concern that placing an external corset which decreases the size of the dilated fragment of the aorta may increase the stress in the aortic wall and lead to a degeneration resembling the process observed in decubitus.

One way of verifying whether a decrease in the diameter of a moderately dilated ascending aorta with an external corset causes any stress increase in the aortic wall is a biomechanical analysis using the finite elements method. This is a computational method, which demonstrates an approximation of the exerted stress that can occur given the necessary entry data of the object and its surroundings (force, movement, material, shape).

The aim of this study was to assess the stress distribution in the aortic wall following a wrapping procedure. A biomechanical analysis comparing wrapping with a dilated and nondilated aorta using the finite elements method was performed.

## Methods

### Finite elements models

Three computational models were created for further analysis – models of the normal aorta and a moderately dilated aorta with and without wrapping. The mechanical properties of the aortic wall were established according to data available from other biomechanical studies [12–14]. Young’s modulus for the aortic wall was 6 MPa and Poisson’s ratio was 0.49. Afterwards, discrete models (divided into finite elements) were created and a sensitivity study of the finite elements’ mesh was carried out. Hexahedral finite elements were chosen for the discretization with a quadratic shape function. The discrete model of aortic wrapping is shown in Fig. 1.

**Fig. 2:**
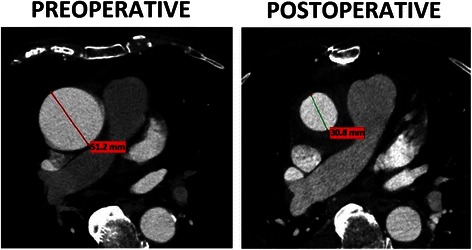
The numerical model of the aorta with the fixation points, axes and the direction of the movement of the aortic annulus

The diameters of the segments of the model of aorta were as follows: 38 mm at the level of the aortic root, 32 mm at the level of the tubular ascending aorta (45 mm in the model of the moderately dilated aorta) and 28 mm at the level of the descending aorta. The radius of the lesser curvature of the aortic arch was 25 mm. The diameters of the main branches of the aortic arch were 10 mm. In the wrapping technique, the aorta is additionally covered by the vascular prosthesis. In our experience, the aortic wall of the moderately dilated ascending aorta (40–55 mm) does not plicate when its diameter is decreased to approximately 30 mm (Fig. 2). Therefore, the inner surface of the wrapped portion of the aorta was simulated as an even surface.

### Simulation

**Fig. 3:**
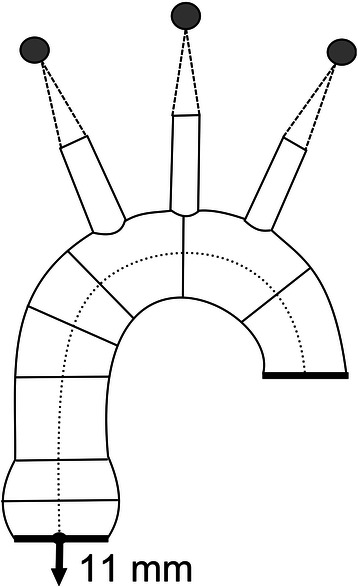
A comparison of the preoperative and postoperative angio-CT images of the ascending aorta subjected to an external wrapping

The aorta was subjected to a pulsatile flow. The analyses were carried out during a single cardiac cycle with a systolic pressure of 120 mmHg and a diastolic pressure of 80 mmHg. The heart cycle was divided into 12 phases and the stress values for every phase were obtained. Systolic up-and-down movements of the proximal ascending aorta were also taken into account. An 11 mm systolic up-and-down motion of the aortic annulus was applied in all simulations (Fig. 3). The branches of the aortic arch were virtually suspended to allow stretching of the ascending aorta during systole and the movement on the Y axis. The distal part of the model, which corresponds to the proximal part of the descending aorta, was immobilized so that it could only be rotated around the Z axis. The tissues around the aorta were not simulated.

In this study, the stress distribution on the outer and inner surface of the aorta was analyzed. It was assumed that the stress distribution on the inner surface might correlate with the potential risk of aortic dissection.

Boundary conditions (arterial pressure, aortic annulus dislocation) were identical in all the simulations. The dimensions of the analyzed models were the only variables. The simulations were carried out using Ansys software (Ansys, Inc.).

## Results

The highest values of stress in the aortic wall were observed at the end of the systole, when arterial pressure was at its peak and the heart muscle was completely contracted and maximally pulled the aortic annulus. All stress values presented in this study were recorded during the above mentioned phase of the heart cycle.

**Fig. 4:**
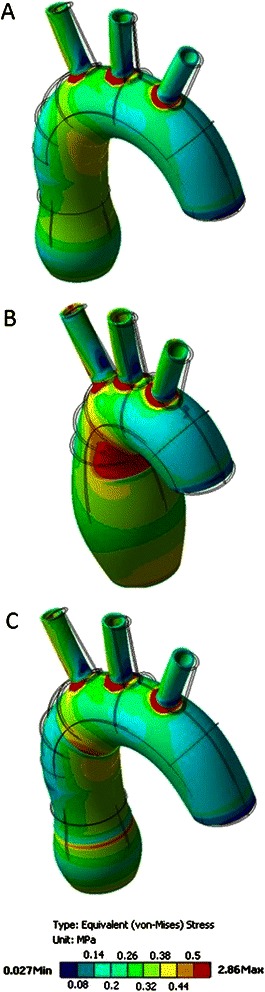
Stress distribution on the outer surface of the aorta. **a** – normal aorta, (**b**) – aortic aneurysm, (**c**) –external wrapping

The distribution of stress on the outer surface of the ascending aorta which was most similar to that observed in the normal aorta (0.03–0.7 MPa) was seen in the wrapping model (0.05–0.8 MPa). The highest values of stress in the aortic wrapping model were noted near the margins of the vascular prosthesis. Overall, the highest values of stress were observed in the aneurysm model (0.06–1.4 MPa), especially in the area between the lesser curvature of the aortic arch and the tubular part of the ascending aorta. The graphical visualization of the distribution of stress on the outer surface of the models is shown in Fig. 4.

**Fig. 5:**
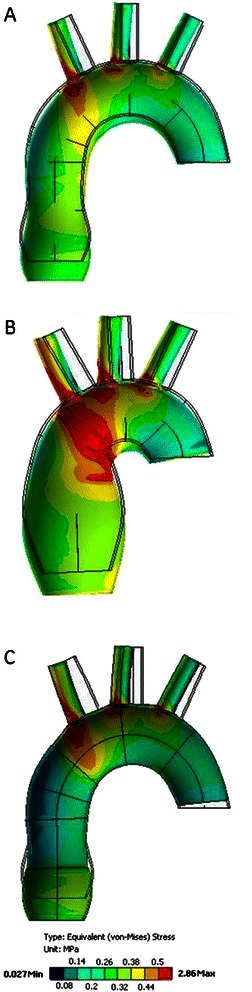
Stress distribution on the inner surface of the aorta. **a** – normal aorta, (**b**) – aortic aneurysm, (**c**) – external wrapping

In all the models, the highest values of stress were noticed in the distal part of the ascending aorta, at the junction with the aortic arch. The highest values of stress on the inner surface of the ascending aorta were observed in the aneurysm model (0.3–1.3 MPa). They ranged from 0.2 MPa to 0.4 MPa in the model of the normal aorta and from 0.05 MPa to 0.4 MPa in the aortic wrapping model. The stress values in the aortic wall underneath the vascular prosthesis in the aortic wrapping model were lower than in the model of the normal aorta. There was a 0.1 MPa difference between the stress values around the edges of the vascular prosthesis in the aortic wrapping model (0.2 MPa in the wrapped portion of the aorta vs. 0.3 MPa in the nonwrapped aorta). The graphical presentation of the stress distribution on the inner surface of the ascending aorta in all models is shown in Fig. 5.

## Discussion

Aortic wrapping is considered to be a controversial surgical method. To date, the results of wrapping with and without concomitant aortoplasty have been promising [9, 15, 16, 10, 17–20, 3, 21–23, 5, 24, 25, 6, 7, 26, 8]. There are no papers, which report increased mortality and morbidity in patients after a wrapping procedure compared to those after a supracoronary graft operation. This surgical technique has the advantage of a shorter aortic cross clamping time, and thus, a potentially lower risk of intraoperative ischemic damage of the myocardium [18, 26, 15] However, there have been documented cases of complications after this procedure [9–11, 27]. The main concern among the adversaries of wrapping is that it may cause the degeneration of the wrapped portion of the aortic wall.

During an isolated wrapping procedure, the diameter of the vessel is decreased without excising its wall. Based on our observations, there are no “wrinkles” or plications of the aortic wall after an isolated wrapping procedure of a moderately dilated aorta. This could result from the residual elasticity of the moderately dilated aorta, which may return to its normal diameter when an external scaffold is applied. One possible explanation is that the arterial pressure pushes the aortic wall against the external scaffold and prevents it from being plicated.

To assess the potential unfavorable biomechanical characteristics of the aorta subjected to a wrapping procedure, the analysis of stress distribution in the aortic wall is needed. Such an analysis should then be compared to the values observed in a “healthy aorta”. One of the best methods of defining the stress distribution is a finite elements analysis (FEA). It involves dividing an analyzed object into numerous smaller elements and carrying out mechanical analyses of the model. The results are an approximation of the phenomena that may occur in the real object. This method is primarily used for engineering purposes. It allows the assessment of the distribution of stress and strain and the investigation of the critical regions within an analyzed object. The finite elements analysis has already been used in cardiovascular medicine [11–19] but there are no studies of stress analyses of the wrapping of the ascending aorta. In our simulations, the highest values of stress in all the analyzed models were observed around the attachments of the main branches of the aortic arch. The branches of the aortic arch had to be virtually suspended (Fig. 3). This implicated the highest stress in the angles between the surface of the aortic arch and its branches. In this study, the results from this area were not analyzed as they were clinically less relevant than the stress analysis in the aortic root and the tubular part of the ascending aorta.

The values of stress on the inner and outer surfaces of the aorta in the model of external wrapping were comparable to the values observed in the normal aorta. Moreover, the values of stress on the inner surface of the wrapped portion of the aorta were even lower than in the nondilated aorta. This means that from a biomechanical point of view, the inner surface of the wrapped aorta is subjected to lower stress than an unwrapped aorta and should be less likely to dissect.

According to the recent data, the aorta mainly dissects before reaching the threshold diameter, which qualifies it for replacement [28]. Thus, a safe and reproducible method of dealing with a moderately dilated aorta would be very useful. Our results suggest that, from a biomechanical point of view, external wrapping may be a reasonable surgical option for dealing with a moderately dilated aorta. However, based on certain case reports, external load caused by the external wrap may cause aortic wall rarefaction and degeneration. Doyle et al. presented a case of a reoperation after aortic valve replacement with a concomitant wrapping procedure due to prosthetic valve dysfunction. The authors observed a rarefaction of the aorta under the Dacron prosthesis with spots where the vascular prosthesis completely replaced the aorta [27]. The patient did not require replacement of the wrapped portion of the aorta as there were no signs of aortic dissection, and the surgeon did not encounter problems when closing the aortotomy. The other possible complication of external wrapping can occur following an improper placement of the external wrap (a vascular prosthesis that is too short or lack of anchoring sutures), which may lead to the dislocation of the prosthesis and subsequent aortic redilatation.

External wrapping of the moderately dilated aorta may be a useful procedure accompanying the aortic valve procedure. However, one should be aware of the possible complications associated with this technique. An improper placement of the prosthesis may cause wrap dislocation and aortic redilatation. Aortic wall degeneration has also been observed in several patients, but it did not lead to aortic dissection in any of the patients.

### Study limitations

Due to technical limitations, the aortic annulus up-and-down motion was simulated without the additional twist that is observed during systole. The simulations were performed on simplified, theoretical models.

## Conclusions

The results of this study suggest that after a wrapping procedure, the aortic wall is subjected to similar stress that occurs in the normal aorta and lower stress than in the nonwrapped, moderately dilated aorta. From a biomechanical point of view, the wrapped aorta is less likely to dissect than a dilated aorta.
